# Supplementary material for: The representational dynamics of task and object processing in humans
Source: eLife. 2018 Jan 31;7:e32816. doi: 10.7554/eLife.32816 (PMC5811210; doi:10.7554/eLife.32816)
Supplement: Transparent reporting form [file elife-32816-transrepform.pdf]

eLIFE
1st Floor
24 Hills Road
Cambridge CB2 1JP, UK
P 01223 855340
W elifesciences.org
T @elife

# *eLife's* transparent reporting form

We encourage authors to provide detailed information *within their submission* to facilitate the interpretation and replication of experiments. Authors can upload supporting documentation to indicate the use of appropriate reporting guidelines for health-related research (see EQUATOR Network), life science research (see the BioSharing Information Resource), or the ARRIVE guidelines for reporting work involving animal research. Where applicable, authors should refer to any relevant reporting standards documents in this form.

If you have any questions, please consult our Journal Policies and/or contact us: editorial@elifesciences.org.

**Sample-size estimation**

- You should state whether an appropriate sample size was computed when the study was being designed
- You should state the statistical method of sample size computation and any required assumptions
- If no explicit power analysis was used, you should describe how you decided what sample (replicate) size (number) to use

Please outline where this information can be found within the submission (e.g., sections or figure legends), or explain why this information doesn't apply to your submission:

Information about the choice of sample size can be found under *Materials and Methods: Participants*. The sample size was chosen based on previous studies employing multivariate decoding of MEG signals (Carlson et al., 2013; Cichy et al., 2014, 2016). The sample of $n$ = 22 was slightly larger than the sample size of those studies (16-20), but took into account that some participants would have to be excluded. The final sample size was $n$ = 17.

**Replicates**

- You should report how often each experiment was performed
- You should include a definition of biological versus technical replication
- The data obtained should be provided and sufficient information should be provided to indicate the number of independent biological and/or technical replicates
- If you encountered any outliers, you should describe how these were handled
- Criteria for exclusion/inclusion of data should be clearly stated
- High-throughput sequence data should be uploaded before submission, with a private link for reviewers provided (these are available from both GEO and ArrayExpress)

Please outline where this information can be found within the submission (e.g., sections or figure legends), or explain why this information doesn't apply to your submission:

As described in *Materials and Methods: Participants*, our study was conducted in $n$ = 22 participants, which constitutes a biological replicate. Five participants were excluded for the following reasons (multiple reasons per participant possible): behavioral performance below 90 % correct (3 participants), excessive artifacts (1 participant), or incomplete or corrupted recordings (2 participants).

eLife Sciences Publications, Ltd is a limited liability non-profit non-stock corporation incorporated in the State of Delaware, USA, with company number 5030732, and is registered in the UK with company number FC030576 and branch number BR015634 at the address 1st Floor, 24 Hills Road, Cambridge CB2 1JP | August 2014

eLIFE
1st Floor
24 Hills Road
Cambridge CB2 1JP, UK
P 01223 855340
W elifesciences.org
T @elife

**Statistical reporting**

- Statistical analysis methods should be described and justified
- Raw data should be presented in figures whenever informative to do so (typically when N per group is less than 10)
- For each experiment, you should identify the statistical tests used, exact values of N, definitions of center, methods of multiple test correction, and dispersion and precision measures (e.g., mean, median, SD, SEM, confidence intervals; and, for the major substantive results, a measure of effect size (e.g., Pearson's r, Cohen's d)
- Report exact p-values wherever possible alongside the summary statistics and 95% confidence intervals. These should be reported for all key questions and not only when the p-value is less than 0.05.

Please outline where this information can be found within the submission (e.g., sections or figure legends), or explain why this information doesn't apply to your submission:

Details about statistical analyses can be found under *Materials and Methods: Statistical Testing.*

Most analyses were conducted at the group-level using a non-parametric, cluster-based statistical approach using the maximum cluster size method (Nichols and Holmes, 2002), searching for significant temporal clusters using a cluster-inducing threshold of $p < 0.05$ and a cluster size threshold of $p < 0.05$. For analyses involving multivariate decoding, significance was determined as the 95$^{th}$ percentile of maximum cluster sizes using a sign-permutation test across participants. For analyses involving model-based MEG-fMRI fusion, significance was determined as the 95$^{th}$ percentile of maximum cluster sizes using a randomization test across columns of the MEG similarity matrix, as suggested by Nili et al. (2014) A Toolbox for Representational Similarity Analysis. PLoS Comput Biol. For the analysis and comparison of MEG peaks, we used bootstrap sampling to calculate 95 % confidence intervals.

All behavioral results were non-significant, with *F*-scores < 1 not requiring report of *p*-values. For time-series data or brain images, reporting of exact *p*-values is not always informative. In addition, calculating exact *p*-values for clusters can be difficult, because it would require recalculating whether the exact same cluster size would survive at a more stringent cluster threshold. For those reasons, we report p-values according to the specified statistical cut-off.

Reporting effect size estimates such as Cohen's *d* are not useful for results of multivariate decoding or representational similarity analysis, as reported in Hebart & Baker (2017) Deconstructing multivariate decoding for the study of brain function. Neuroimage. However, classification accuracy and explained variance ($R^2$) are reported for multivariate decoding analyses and model-based MEG-fMRI fusion analyses, respectively.

(For large datasets, or papers with a very large number of statistical tests, you may upload a single table file with tests, Ns, etc., with reference to sections in the manuscript.)

eLife Sciences Publications, Ltd is a limited liability non-profit non-stock corporation incorporated in the State of Delaware, USA, with company number 5030732, and is registered in the UK with company number FC030576 and branch number BR015634 at the address 1st Floor, 24 Hills Road, Cambridge CB2 1JP | August 2014

**Group allocation**

- Indicate how samples were allocated into experimental groups (in the case of clinical studies, please specify allocation to treatment method); if randomization was used, please also state if restricted randomization was applied
- Indicate if masking was used during group allocation, data collection and/or data analysis

Please outline where this information can be found within the submission (e.g., sections or figure legends), or explain why this information doesn't apply to your submission:

For details, see *Materials and Methods: Experimental Design and Stimuli*. Our study consisted of one group to which participants were randomly allocated. All participants were exposed to all combinations of our experimental factors.

**Additional data files ("source data")**

- We encourage you to upload relevant additional data files, such as numerical data that are represented as a graph in a figure, or as a summary table
- Where provided, these should be in the most useful format, and they can be uploaded as "Source data" files linked to a main figure or table
- Include model definition files including the full list of parameters used
- Include code used for data analysis (e.g., R, MatLab)
- Avoid stating that data files are "available upon request"

Please indicate the figures or tables for which source data files have been provided:

As Source data, we provide all files used for statistical analyses and creation of figures, including Matlab code to analyze them. These include individual MEG decoding matrices, temporal generalization matrices, and both group MEG and group fMRI similarity matrices used for calculation of model-based MEG-fMRI fusion.

eLife Sciences Publications, Ltd is a limited liability non-profit non-stock corporation incorporated in the State of Delaware, USA, with company number 5030732, and is registered in the UK with company number FC030576 and branch number BR015634 at the address 1st Floor, 24 Hills Road, Cambridge CB2 1JP | August 2014
